# Sex chromosome complement and sex steroid signaling underlie sex differences in immunity to respiratory virus infection

**DOI:** 10.3389/fphar.2023.1150282

**Published:** 2023-03-30

**Authors:** Reegan A. J. Miller, Abigael P. Williams, Susan Kovats

**Affiliations:** ^1^ Arthritis and Clinical Immunology Program, Oklahoma Medical Research Foundation, Oklahoma City, OK, United States; ^2^ Department of Microbiology and Immunology, University of Oklahoma Health Sciences Center, Oklahoma City, OK, United States

**Keywords:** respiratory virus, pulmonary immunity, sex differences, estrogen, androgen, X chromosome inactivation

## Abstract

Epidemiological studies have revealed sex differences in the incidence and morbidity of respiratory virus infection in the human population, and often these observations are correlated with sex differences in the quality or magnitude of the immune response. Sex differences in immunity and morbidity also are observed in animal models of respiratory virus infection, suggesting differential dominance of specific immune mechanisms. Emerging research shows intrinsic sex differences in immune cell transcriptomes, epigenomes, and proteomes that may regulate human immunity when challenged by viral infection. Here, we highlight recent research into the role(s) of sex steroids and X chromosome complement in immune cells and describe how these findings provide insight into immunity during respiratory virus infection. We focus on the regulation of innate and adaptive immune cells by receptors for androgen and estrogens, as well as genes with a propensity to escape X chromosome inactivation. A deeper mechanistic knowledge of these pathways will help us to understand the often significant sex differences in immunity to endemic or pandemic respiratory pathogens such as influenza viruses, respiratory syncytial viruses and pathogenic coronaviruses.

## Introduction

Respiratory virus infections present a global health burden. During the 2019–2020 influenza season, nearly 35 million cases and over 20,000 deaths occurred in the United States alone, with approximately $11.2 billion spent on healthcare (CDC, 2021). The COVID-19 pandemic continues to increase the numbers of respiratory infection cases. Epidemiological studies have shown human sex differences in infectious disease incidence, immune response quality, and treatment response, and in immunity upon vaccination against pathogens [reviewed in (Ursin and Klein, 2021; Wilkinson et al., 2022)]. While differential outcomes in respiratory immunity may be explained by cultural factors such as gender disparate workplaces, smoking history, or social habits, sex differences in immunity and morbidity also are observed in animal models of respiratory infection [reviewed in (Klein et al., 2012; Kadel and Kovats, 2018)]. These findings with rodent models suggest that many sex differences arise through biological mechanisms influenced by sex chromosome complement and/or sex steroid levels. Gender and sex differences in morbidity and/or immunity often present after the onset of sexual maturity, suggesting a role for higher levels of estrogens or androgens (Jacobsen and Klein, 2021). Investigation of pathways through which divergent sex steroid levels or sex steroid receptor activity influence overall morbidity and the immune cell-specific response to infectious disease has led to important new insights as detailed below. In addition, emerging research has shown that immunity is regulated by sex chromosome complement and the gene dosage effect resulting from escape of specific genes from X chromosome inactivation (Jiwrajka and Anguera, 2022). Mechanistically, the pathways downstream of sex steroid receptor activity may work in tandem with sex chromosome complement to influence the immune response to respiratory pathogens. Investigation of these pathways will increase our understanding of infectious disease pathogenesis and inform better treatment options based on biological sex and gender Box 1.

BOX 1 Sex and gender. Sex and gender are distinct terms. While sex has historically been considered a strict binary variable, new information reveals that manifestation of biological sex is influenced by chromosomal complement, genetics, including alleles leading to disorders of sex development (DSDs), sex steroid concentrations and external/internal anatomy [discussed in (Ainsworth, 2015; Jacobsen and Klein, 2021)]. Gender is a social construct that does not exist in a binary, and its external manifestation may include manipulation of sex hormone levels, particularly by transgender and gender non-conforming individuals. In humans, sex steroid levels may vary greatly with health status, age (including milestones such as puberty, pregnancy, or menopause and andropause), and/or direct manipulation of levels through contraceptives, hormone replacement therapy, and/or gender affirming hormone treatments. Failure to measure and report these sex steroid levels in studies of humans inhibits our understanding of the mechanistic basis of observed sex differences in immune cell numbers or activation pathways. Thus, studies of human immunity will benefit from ascertainment of the gender identity, assigned sex at birth, sex chromosome complement, and sex steroid level of individuals, and integration of these variables into analyses of immune parameters and disease outcomes. Encouragingly, an increasing number of human studies do report gender and/or sex as a non-binary variable or include measurements of circulating sex steroid levels; for example (Furman et al., 2014; Ter Horst et al., 2016; Webb et al., 2018; Robinson et al., 2022). Here, unless otherwise noted, we refer to sex differences when discussing human population studies, as most studies report participants as male or female using definitions set by the study authors.

### From the clinic: Epidemiological evidence for gender differences in the incidence and pathogenesis of respiratory infection

The incidence, morbidity and immune response to acute respiratory virus infection vary with sex and age. For example, sex differences in influenza incidence and severity depend on the age group, with increased morbidity and mortality associated with young (0–20 years) and elderly (>69 years) males, yet associated with females during reproductive age (20–49 years) (Kumar et al., 2009; WHO, 2010; Eshima et al., 2011; Hoffmann et al., 2015; Giurgea et al., 2022). Consistent with this, another study showed that the frequency of early life hospitalization for severe lower respiratory tract infection or bronchiolitis was elevated in male compared to female children (Regis et al., 2021). Infant and older adult males show increased incidence and/or severity of respiratory syncytial virus infection of the lower respiratory tract (De Jacobis et al., 2020; Orimadegun et al., 2020; Branche et al., 2022; Vila et al., 2022).

Epidemiological data show that males 45–79 years of age suffered greater morbidity and mortality when infected with pathogenic coronaviruses, such as SARS-CoV-1, MERS, and SARS-CoV-2 (Karlberg et al., 2004; Alghamdi et al., 2014; Gebhard et al., 2020; Alwani et al., 2021; Gomez et al., 2021; Scully et al., 2021). During the COVID-19 pandemic, reports of increased morbidity and mortality in elderly males have fueled new research in sex differences in immunity to infectious pathogens. Recent reports detail sex differences in immune system cells and related proteins in COVID-19 patients, and in some cases, these immune differences have been linked to sex-specific outcomes in morbidity or mortality (Takahashi et al., 2020; Hou et al., 2021; Ren et al., 2021; Butler-Laporte et al., 2022).

An open question is the extent to which biological sex (with inherent sex steroid levels and chromosome complement) shapes an individual’s intrinsic capacity for immune responses, and several recent studies provide evidence for this idea. Identification of innate immune endotypes predicting antibody responses through a pan-vaccine analysis revealed that biological sex and age explained small fractions of the variance in transcriptomic data collected (Fourati et al., 2022), suggesting that stratification of these types of data by biological sex may elucidate sex-divergent pathways in the human population. A study of transcriptional, epigenetic, and cellular changes in the human immune system revealed a sexual dimorphism in the profile of aging in peripheral blood mononuclear cells (PBMCs) (Marquez et al., 2020). The data, obtained using RNA-seq, ATAC-seq, and flow cytometry analyses of healthy adult men and women aged 22–93, showed an epigenomic signature of aging characterized by declining naïve T-cell functions and increased monocyte and cytotoxic lymphocyte functions. Genomic differences between sexes increased after age 65, with men showing acceleration of the aging signature, including higher innate and pro-inflammatory activity and reduced adaptive activity. These findings have implications for the quality of antiviral responses and may help to explain the increased morbidity often observed in elderly men. Another study showed that healthy younger (< age 50) males and elderly (> age 60) individuals of both sexes showed differences in immune cell proportions in blood and levels of circulating mediators that tend to correlate with the profile observed in severe COVID-19, suggesting both sex and age dependent immune variables that increase susceptibility to severe infectious disease (Kilic et al., 2021). Characterization of whole-genome autosomal DNA methylation in men and women aged 32–81 showed substantial differences that correlated with differential gene expression, thus defining sex-specific DNA methylation patterns that likely mechanistically underpin sex differences in immunity (Singmann et al., 2015). These findings are consistent with sex differences in inflammation and trained immunity upon BCG vaccination (Koeken et al., 2020).

Going forward, clinical data disaggregated by gender, sex, and age will inform our understanding of how biological sex and gender influence the incidence and pathogenesis of infectious disease. A barrier to studying human responses to infectious agents is the frequent absence of early ascertainment and sampling. For more common outbreaks, such as seasonal influenza, individuals often do not seek treatment for mild disease, which skews the sex outcome data towards more severe cases and hospitalizations. In the COVID-19 pandemic, early detection and reporting of SARS-CoV-2 infections has increased our understanding of the course of the disease, as well as how sex and gender may influence antiviral immunity and pathogenesis.

### Sex steroid hormones and their receptors

Endogenous estrogens include estrone (E1), 17-β-estradiol (E2), and estriol (E3), with E2 predominant in adult females and males, albeit at distinct levels, and E3 elevated in pregnancy. Testosterone is synthesized in males and females and converted by 5α-reductase to the physiologically active dihydrotestosterone or by aromatase to E2 (Sathish et al., 2015). Sex steroids are synthesized in the gonads and adrenal cortex as well as peripheral tissues such as fat or liver (Labrie et al., 2005; Sathish et al., 2015; Huang et al., 2021). Activated lung macrophages expressing aromatase may regulate local E2 levels, but more information is needed about how this is impacted by respiratory infection (Sathish et al., 2015). In humans, levels of sex steroids vary with age (puberty, menopause, andropause) and/or due to external manipulation such as by contraceptives and/or other exogenous hormone therapy. While the major sex steroid surge is at puberty, neonatal males are exposed to high levels of testosterone in a minipuberty stage (Quigley, 2002; Renault et al., 2020). Furthermore, infection or inflammation can temporarily alter levels of androgens and estrogens (Robinson et al., 2011b; Vom Steeg et al., 2019; Tuku et al., 2020). Endocrine disrupting chemicals (EDCs), including phenols, parabens, and phthalates, also have been reported to modulate innate and adaptive immune function, although precise effects are difficult to ascertain since EDCs bioaccumulate over time (Popescu et al., 2021). EDCs are often partial estrogen receptor (ER) agonists that may modulate ER-mediated transcriptional or rapid signaling pathways. Thus, EDCs may have greater effects in females due to their to competition with endogenous estrogens for ER binding.

The sex steroid receptors for estrogens (ERα encoded by *Esr1* and ERβ encoded by *Esr2*) and androgen (AR encoded by *Ar*) act as ligand-dependent transcription factors. Upon binding ligand in the cytoplasm, the receptor/ligand complex translocates to the nucleus, and, guided by the presence of pioneer factors such as FOXA1, binds to specific androgen or estrogen response elements (ARE or ERE, respectively) located at target genes. Once bound, recruitment of co-activators and/or repressors allows for modulation of gene transcription through epigenetic changes, such as modification of histone methylation or acetylation, leading to chromatin remodeling (Mann et al., 2011; Pihlajamaa et al., 2015; Liu et al., 2017). The expression of androgen or estrogen receptor RNA or protein on various immune cell types is summarized in (Kadel and Kovats, 2018) and is available in the Immunological Genome Project [Immgen.org] (Heng et al., 2008). For example, macrophages generally express *Esr1* and *Ar*, while type 2 innate lymphocytes primarily express *Ar*. Levels of AR or ER expression may increase or decrease with cellular activation, as shown for AR in T cells (Guan et al., 2022). Therefore, the magnitude and/or complement of ER/AR expression on resident or infiltrating immune cells in the lung is likely to regulate their patterns of gene expression and influence functional responses. Furthermore, ER or AR signaling in hematopoietic progenitors may set up epigenetic patterns that are preserved in descendent immune cells (Igarashi et al., 2001; Carreras et al., 2008). Murine models to study mechanisms underlying sex differences are described in Box 2.

Sex steroids influence the course of disease through modulation of the overall inflammatory environment and through direct regulation of immune cells. Androgens are largely considered anti-inflammatory (Gilliver, 2010; Cutolo and Straub, 2020), while estrogens, such as E2, elicit both pro- and anti-inflammatory effects, depending on levels of sex steroids *in vivo* or in cultured cell experiments (Kovats, 2015; Cutolo and Straub, 2020). For example, murine gonadectomy with or without hormone replacement prior to influenza A virus (IAV) infection has shown that testosterone is protective in sublethal infections (Vermillion et al., 2018b; Vom Steeg et al., 2020). High levels of E2 in female mice were protective against lethal infection, which was associated with reduced levels of pro-inflammatory cytokines (Robinson et al., 2011b). Estrus-diestrus levels of E2 promote inflammatory DC differentiation by upregulating IRF4 in myeloid progenitors (Carreras et al., 2010). However, despite these advances in our understanding, differing levels of sex steroids in the human population have not been conclusively linked with infection outcome in the COVID-19 pandemic [reviewed in (Lott et al., 2022)].

Biological sex also may regulate other variables influencing pulmonary immunity. Sex differences in lung architecture secondary to sex steroid levels may contribute to differential immunity to respiratory virus infection (Carey et al., 2007). Sex-specific expression levels of viral entry receptors, such as ACE2 or TMPRSS2 for SARS-CoV-2, may impact the initiation or magnitude of infection (Lott et al., 2022).

### Incomplete and variable X chromosome inactivation (XCI)

X chromosome inactivation (XCI) occurs early in embryonic development to compensate for the gene dosage imbalance resulting from the additional X chromosome(s) in females (Souyris et al., 2019; Jiwrajka and Anguera, 2022). Once XCI is initiated, X-inactivation specific transcript (XIST/Xist), a long non-coding RNA, is upregulated on the randomly chosen, future inactive X chromosome (Xi). The transcribed XIST/Xist RNA accumulates and condenses on the X chromosome, recruiting other complexes to epigenetically silence the chromosome. XCI is not permanent, and XIST must be continuously transcribed to maintain the inactivated state. However, not all genes on the Xi are silenced, as an estimated 15%–20% of genes on the X chromosome escape inactivation, with an additional 10%–12% displaying variable escape between individuals (Wang et al., 2016; Syrett and Anguera, 2019).

The X chromosome has the highest density of immune response genes, and many are known to escape XCI in humans or mice, such as *TLR7*, *CXCR3*, *CD40LG*, *BTK, IRAK1, NEMO* and *CXORF21* (Wang et al., 2016; Souyris et al., 2018; Harris et al., 2019; Odhams et al., 2019). Although escape from XCI leads to biallelic expression of these genes, thus altering the gene-dose effect, it is not the only factor that contributes to altered expression of X-linked immune genes. Maintenance of the XIST RNA is variable during activation of lymphocyte populations (Wang et al., 2016). In naïve lymphocytes, the Xi is partially reactivated *via* decreased maintenance of the XIST RNA, and this phenotype changes to full inactivation upon lymphocyte interaction with antigen and subsequent functional activation. This transition from partial reactivation to fully inactive contributes to the differences in biallelic gene expression observed in naïve *versus* activated lymphocytes. While the contributions of X chromosome complement (including aneuploidy), XCI, and escape from XCI have been primarily studied in the context of autoimmunity, these findings have implications for antiviral immunity (Abramowitz et al., 2014).

BOX 2 Murine models to study mechanisms underlying sex differences. Murine models to study the influence of sex steroids range from simple physiological manipulations to global or conditional disruption of *Esr* or *Ar* genes. Gonadectomy with or without hormone replacement is often used to study the role of sex steroids in infectious disease outcome and immune responses [for example (Laffont et al., 2014; Vom Steeg et al., 2016; Laffont et al., 2017; Vermillion et al., 2018a)]. Mice with global *Esr1* deficiency have been used to investigate the role of ERα activity; however, these data are often confounded by the abnormally high levels of testosterone in both female and male *Esr1* ^*−/−*^ mice, as well as high levels of estradiol in female mice (Sims et al., 2002). AR^*Tfm*^ (testicular feminization) mice, with a spontaneous mutation in the *Ar* gene that leads to androgen insensitivity, have been used to understand how a global absence of AR activity modulates immune responses (He et al., 1991); yet a potential caveat to such studies is that *Tfm* mice have low levels of serum testosterone (Jones et al., 2003), which may lead to changes in estradiol levels. While these approaches increase understanding of the systemic impact of sex steroids, they do not identify cell intrinsic effects of ER or AR activity. Models that delete sex steroid receptor genes in specific immune cell types have elucidated ER or AR mediated cell intrinsic mechanisms without disruption of systemic hormone levels or synthesis pathways (Griesbeck et al., 2015; Casali et al., 2020; Ejima et al., 2022). A unique mouse model that enables assessment of the roles of sex chromosomes and sex steroids separately is the four core genotypes (FCG) model. In FCG mice, the testis-determining *Sry* gene is located on an autosome, so that XX and XY mice can either be gonadally male (express SRY) or female (do not express SRY). With the four genotypes available (XXF, XYF, XXM, XYM), comparison of these mice determines if sex differences are a result of gonads/sex steroids, sex chromosomes, or a combination of both (Arnold and Chen, 2009; Robinson et al., 2011a).

**TABLE 1:** Pathways shown in Figure 1.

Key	Pathway	Sex difference or sex steroid regulation or XCI effect
A	ssRNA virus damages lung epithelium	• Sex differences in expression of viral entry receptors
B	Damaged epithelium releases chemokines, alarmins and cytokines to recruit or activate innate immune cells such as DCs, monocytes, neutrophils and ILCs/NKs	• Sex differences in IL-33 production were reported
		• AR promotes development of IL-33 producing stromal cells (in visceral adipose tissue)
C1	ssRNA activates TLR7-TASL signaling that results in production of IFN-I and proinflammatory cytokines	• ERα activity promotes pathways involving *IRF5* leading to increased IFN-I
		• X-linked *TLR7* and *CXORF21* expression is increased by incomplete XCI in females
C2	Activated ILC2s produce IL-5 that recruits eosinophils and amphiregulin that promotes tissue repair	• Females harbor more ILC2s than males
		• ILC2 numbers and function are attenuated by AR activity
		• Overall amphiregulin levels are increased in males and by AR activity
D	Activated cDCs transit to LNs and present Ag to naïve T cells. Inflammatory monocyte-derived DCs infiltrate lung tissue	• ER activity regulates DC differentiation and function
E1	Activated CD8^+^ T cells differentiate to cytotoxic effectors or memory cells	• AR activity promotes faster contraction of CTL after viral clearance, promoting faster recovery
		• AR activity limits IFNγ production
E2	CD4^+^ T cells differentiate to Th1 cells that produce IFNγ and promote viral clearance	• X-linked *CXCR3* subject to incomplete XCI leading to biallelic expression in females
		• T cell CXCR3 promotes antiviral activity
		• ER activity promotes CD4^+^ T cells functional responses
		• AR activity reduces Th1 differentiation
E3	CD4^+^ T cells promote B cell activation and Ab production	• X-linked *CD40LG* subject to incomplete XCI leading to biallelic expression in females
		• CD40L enhances T-B interactions and T cell survival
E4	Regulatory T cells attenuate immune responses and promote CD8^+^ T memory cell differentiation	• ER activity promotes FOXP3 expression and regulatory T cell activity
		• AR activity may promote regulatory T cell numbers and amphiregulin production during the resolution phase
F	B cells produce Abs specific for viral proteins	• Biallelic TLR7 expression due to incomplete XCI increases quantity and quality of virus-specific Abs
		• ERα activity promotes class switch recombination and somatic hypermutation *via* effects on BCR signaling and *Aicda* expression
		• AR activity decreases Ab levels

## Sex differences in innate antiviral immunity

**FIGURE 1:**
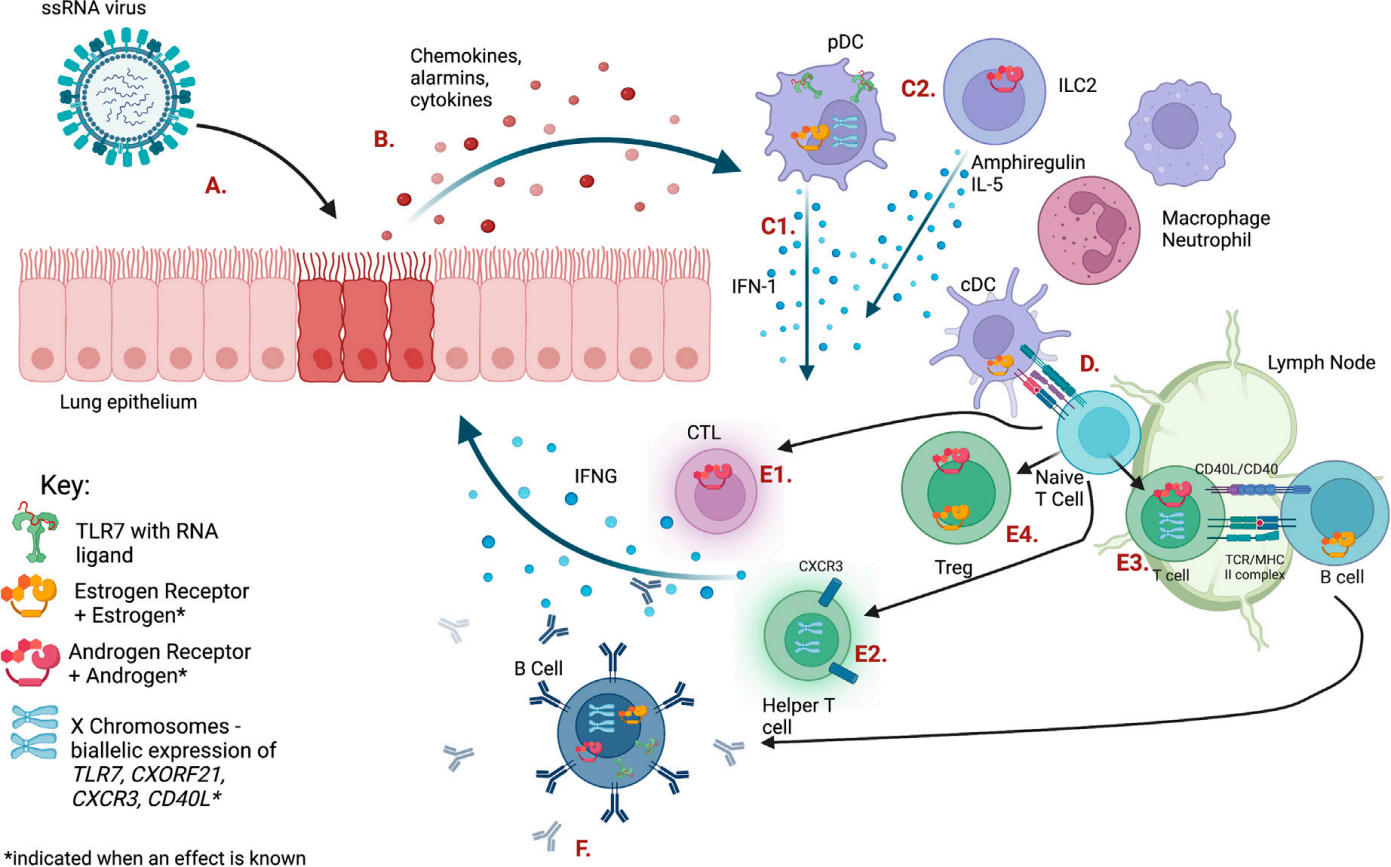
The immune response to ssRNA viral infection is regulated by sex steroid receptors and gene dosage on the X chromosome. Letters indicating each cell type or pathway are defined in Table 1. *Figure created with BioRender.com.*

Upon infection with a single-stranded RNA (ssRNA) virus, such as respiratory syncytial virus (RSV), influenza A or B virus (IAV, IBV), or SARS-CoV-2, cells within the respiratory tract initiate the immune response by producing antiviral molecules and proinflammatory cytokines (Mettelman et al., 2022). Type I interferons (IFN-I) are produced after ssRNA recognition and signaling *via* endosomal TLR7 and other cytosolic nucleic acid sensors, such as MDA5 (Diebold et al., 2004; Okude et al., 2020). IFN-I stimulates the transcription of numerous genes involved in both viral clearance and enhancement of the innate immune response (Schoggins and Rice, 2011). IFN-I is initially produced by pulmonary epithelial cells as well as lung resident macrophages and conventional dendritic cells (cDCs), which also secrete chemotactic molecules to quickly recruit other innate immune cells from blood, such as neutrophils, monocytes, natural killer (NK) cells, and plasmacytoid dendritic cells (pDCs) (Figure 1, Table 1) (Mettelman et al., 2022).

Recent clinical studies show that females have an increased propensity to generate robust antiviral responses involving IFN-I. Stimulation of PBMCs from male and female teenagers with live virus, or TLR7 or TLR9 stimuli, showed that females produced higher levels of IFN-I, IFNγ and IFN-induced chemokines compared to males (Regis et al., 2021). A recent report on sex differences in SARS-CoV-2 infection in young military recruits followed longitudinally showed that females had higher pre- and post-infection expression of antiviral interferon-stimulated genes (ISGs) (Sauerwald et al., 2022), which correlated with increased illness symptoms, yet reduced viral load. A heightened state of antiviral innate immunity prior to infection in females is consistent with our understanding of sex-specific mechanisms regulating IFN-I pathways, as outlined below.

**FIGURE 2:**
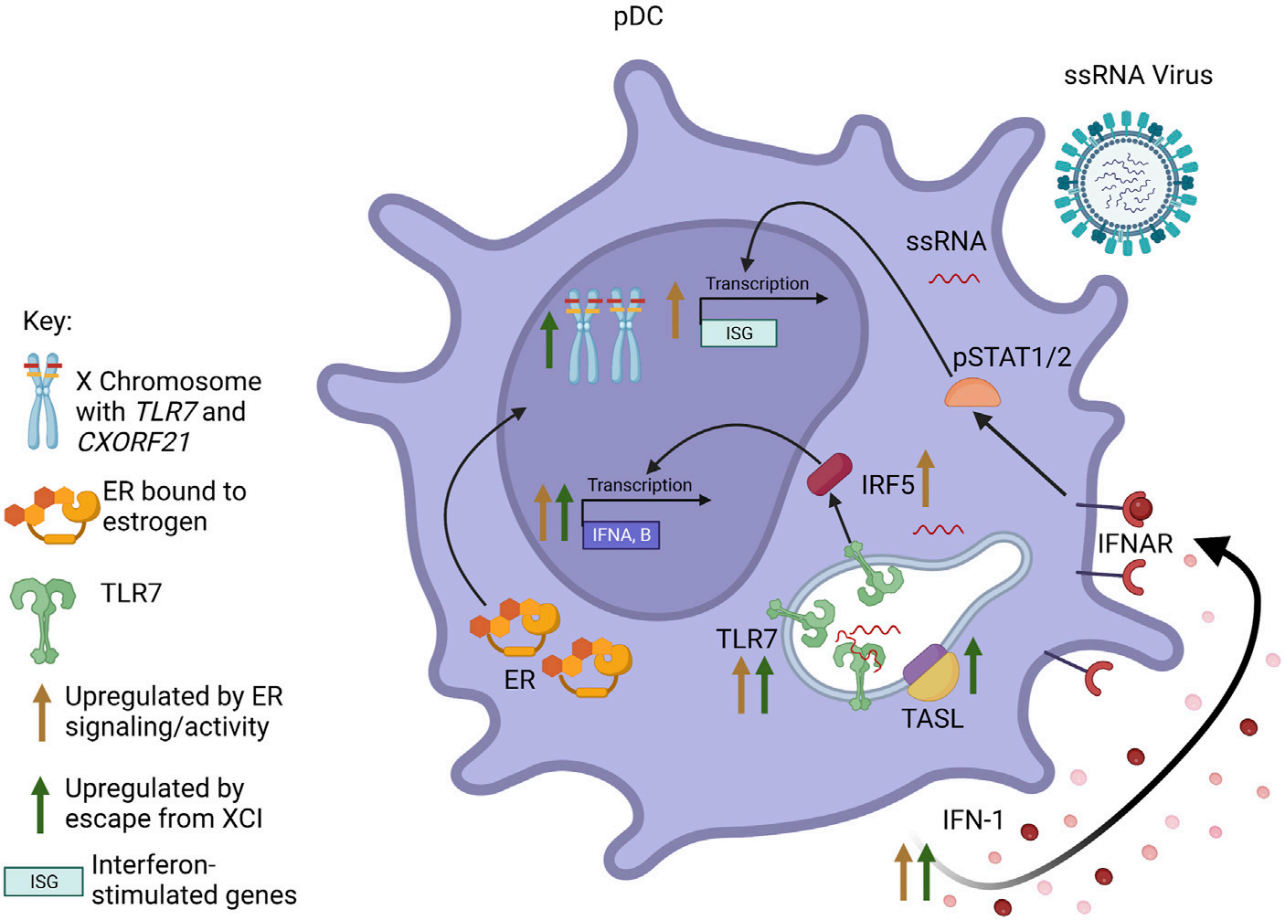
Interaction between sex steroid receptors and escape from XCI in pDCs. Signaling through ER results in increased pDC responsiveness to TLR7 signaling and TLR7-mediated IFN-I production. Additionally, ER activity increases transcription of IFNA/B and interferon-responsive genes (ISGs), both through increased production of IFN-I leading to signaling through the IFNAR, as well as increased expression of IRF5. Escape from XCI by *TLR7* and *CXORF21* results in biallelic transcription of the genes. Increased levels of TLR7 and TASL (the protein encoded by *CXORF21*) also result in increased transcription of IFNA/B and increased production of IFN-I. *Figure created with BioRender.com.*

A body of evidence shows that estradiol acting *via* ERα promotes the production of IFN-I, often by regulation of genes in innate sensing pathways such as *TLR8*, *Unc93b1*, *Aim2, Trim21* or *Irf5,* whose activity leads to the induction of the *Ifna* or *Ifnb* genes [reviewed in (Kovats, 2015)]. X linked genes, such as *TLR7*, that escape dosage compensation also contribute to increased IFN-I production in female cells. These mechanisms lead to sex differences in production of IFN-I or IFN-responsive pathways (Figure 2), which has important implications for antiviral responses, as recently reviewed (Pujantell and Altfeld, 2022).

Human and murine pDCs express high levels of both *ESR1* and *ESR2* (Phiel et al., 2005; Laffont et al., 2014). pDCs rapidly produce significant amounts of IFN-I in response to ssRNA viruses. Mouse models show that estrogens enhance the responsiveness of pDCs to TLR7 and TLR9 signaling, and treatment of postmenopausal women with estradiol restored cytokine production specifically in pDCs, indicating a cell-specific mechanism (Seillet et al., 2012). A later study using mice reconstituted with human CD34^+^ cells from cord blood showed this enhancement of TLR7-induced IFN-I production to be dependent on estrogen in the host, regardless of the sex of the donor, although X chromosome dosage independently contributed to the sex bias (Laffont et al., 2014). *Esr1* deficiency restricted to either the hematopoietic compartment or DCs in mice revealed that the enhanced TLR7 signaling in female pDCs is likely due to direct regulation of *IRF5* expression by ERα (Griesbeck et al., 2015). Increased basal *IRF5* expression perpetuated by ERα signaling in pDCs, and perhaps other cells, may contribute to the elevated IRF5-dependent IFN-I production observed in females compared to males.

The sex differences in pDCs also are influenced by sex chromosome complement (Souyris et al., 2018). *TLR7* is encoded on the X chromosome and is confirmed to escape XCI in immune cells, including monocytes and pDCs, from both female (46, XX) and Klinefelter syndrome (KS) male (47, XXY) individuals. Stimulated PBMCs from women and KS men produced similar amounts of *TLR7* mRNA, which was greater than that produced by PBMCs from (46, XY) men and (45, XO) Turner syndrome (TS) women (Sarmiento et al., 2019). Furthermore, within PBMCs treated with a TLR7 agonist, pDCs from (46, XX) women and (46, XX) transgender men produced more type I IFN and had higher expression of CD86 (a surface marker of pDC activation) than (46, XY) men and (46, XY) transgender women, indicating the sex difference seen was more dependent on sex chromosome complement than sex steroid concentration (Webb et al., 2018). A recent study of COVID-19 patients showed that while *TLR7* is expressed similarly in non-ICU patients of both sexes, *TLR7* expression in blood cells is significantly downregulated in male patients admitted to the ICU as compared to female patients (Gomez-Carballa et al., 2022). Additionally, analysis of DNA methylation patterns on the *TLR7* gene revealed four differentially methylated positions that were significantly different between men with severe and mild COVID-19, whereas there were no significant *TLR7* methylation differences between females. The reduced *TLR7* expression in males with severe disease suggests that these individuals had decreased early antiviral responses.

As IFN-I is critical for the initiation of the antiviral immune response, sex divergent production kinetics could lead to differences in disease outcome. Studies using mouse models of SARS-CoV-1 indicate that a delayed IFN-I response contributes to immunopathology, as the initial paucity of IFN-I and increased viral load leads to the recruitment of inflammatory macrophage-monocytes (IMM) and neutrophils into the lungs of infected mice (Channappanavar et al., 2016). In both MERS-CoV and IAV infections, the timing of the IFN-I response is vital to viral clearance, as early treatment with either rIFNβ or rIFNɑ provided total protection, while delayed treatment after the peak of viral replication exacerbated disease (Davidson et al., 2016; Channappanavar et al., 2019). This scenario also likely occurs upon SARS-CoV-2 infection in humans (Sette and Crotty, 2021). In the murine model of sublethal SARS-CoV-1 infection, males were more likely to show significant morbidity and mortality compared to females, particularly when older (8–20 months) (Channappanavar et al., 2017). While viral titers were higher in males, levels of *Ifnb* RNA were comparable. However, males showed increased levels of proinflammatory mediators (CCL2, CXCL1, IL-6) and lung infiltration of IMMs and neutrophils, suggesting a more exuberant inflammatory response and perhaps altered kinetics of IFN-I production or accumulation. Ovariectomy and the ER antagonist ICI182,780 decreased survival. These experiments showed that ER signaling protects the female mice during infection by decreasing IMM recruitment and viral load. In contrast, orchiectomy, or treatment with the AR antagonist flutamide did not alter morbidity or mortality in males.

## Sex differences in lymphocyte mediated immunity

Lymphocyte immunity involves innate lymphocytes producing cytokines or other cytotoxic mediators (e.g., ILCs, NK or MAIT cells), or B and T cells implementing antigen-specific adaptive immunity. After acquiring viral antigens at the site of viral infection, DCs migrate to the draining lymph nodes where they present antigen to naïve T cells (Mettelman et al., 2022). Naïve T cells differentiate into cytotoxic (CD8^+^) or helper (CD4^+^) subsets based on peptide recognition and/or environmental cytokines. During respiratory viral infections, CD8^+^ cytotoxic T cells promote viral clearance through cytokine release (IFN𝛄, TNFα, IL-2), degranulation, and contact-dependent apoptosis of infected cells. CD4^+^ T helper and regulatory cells release cytokines that recruit innate immune cells, regulate CD8^+^ T cell activity, suppress the inflammatory environment, and promote B cell differentiation and antibody production. As T cell responses are vital to the immune response, dysregulation in numbers and/or function can negatively impact disease outcome.

Adaptive T and B cell responses in COVID-19 have been extensively reviewed elsewhere, for example in (Sette and Crotty, 2021). The majority of studies on T cells in COVID-19 patients did not report significant sex differences in the extent of T cell activation or numbers of antigen-specific T cells; however, a few studies described here did define differences that could be followed up. Among COVID-19 patients, males harbored a lower CD4^+^:CD8^+^ T cell ratio (Zhao et al., 2021). A decrease in activated T cells was correlated to increased age and mortality in male, but not female, patients with severe disease (Takahashi et al., 2020; Zhao et al., 2021). To date T cell epitope mapping studies have not revealed sex differences in epitope selection (Sette and Crotty, 2021).

Sex differences in the total number and activity of mucosal-associated invariant T (MAIT) cells also correlate with COVID-19 severity of infection (Yu et al., 2021). MAIT cells respond to viral infection through the release of IFNγ, TNFα, and the cytotoxic molecules perforin and granzyme B. Analyses of T cell populations of COVID-19 patients revealed that female patients had a lower proportion of MAIT cells in the blood, but higher numbers in the bronchoalveolar lavage fluid, indicating that MAIT cells may better extravasate to the pulmonary site of infection in females. MAIT cells in females showed a more immunologically active transcriptome, while MAIT cells in males showed an exhausted, pro-apoptotic phenotype, possibly contributing to the increase in immunopathology observed in males.

Sex steroids acting *via* ER or AR elicit both direct and indirect effects on CD4^+^ and CD8^+^ T cell function in diverse models of infection, autoimmunity and cancer. IAV infection of gonadectomized males showed that testosterone causes earlier contraction of IAV-specific IFN𝛄^+^CD8^+^ T cells in the lung after viral clearance, thereby reducing the overall inflammatory environment and increasing the rate of recovery, although this has not yet been linked to T cell intrinsic AR expression (Vom Steeg et al., 2020). Testosterone exposure led to direct binding of AR to the phosphatase gene *Ptpn1,* and the resulting increased transcription of *Ptpn1* led to inhibition of IL-12 induced STAT4 phosphorylation and Th1 differentiation (Kissick et al., 2014). These studies are consistent with new reports showing that AR activity limits T cell effector function and promotes T cell exhaustion in solid tumors. AR bound directly to the *Ifng* gene, and blockade of AR activity or deletion of *Ar* in T cells led to increased IFN𝛄 production by CD8^+^ T cells (Guan et al., 2022; Kwon et al., 2022). Thus, AR activity may limit functional responses of activated antiviral T cells.

In contrast, multiple reports show that ER activity promotes T cell functional responses. ERα action in T cells induces their activation and proliferation in an autoimmune colitis model (Mohammad et al., 2018). Disruption of ERα signaling in a globally deficient *Esr1* ^*−/−*^ murine lupus model led to attenuated T cell activation, loss of spontaneous germinal centers, and loss of anti-chromatin autoantibodies, suggesting ERα regulates lymphocyte activation in this model, although a role for elevated testosterone in the *Esr1* ^*−/−*^ mice cannot be excluded (Graham et al., 2020). Healthy PBMCs from women expressed higher levels (compared to men) of the *CD2* gene, and CD2 expression was upregulated by E2 exposure *in vitro* (Fernandez Lahore et al., 2021). ER-mediated regulation of the costimulatory molecule CD2 governed T cell activation in murine autoimmune models, in which female specific differences in CD2 expression on CD4^+^ T cells were attributed to the action of E2 and the binding of ERα to a polymorphic estrogen receptor binding site near the *Cd2* gene (Fernandez Lahore et al., 2021). Sex differences in the capacity of human and murine CD4^+^ T cells to proliferate and produce IFNγ upon TCR stimulation was linked to levels of PPARα, which represses NF-κB and c-JUN important for T cell activation (Dunn et al., 2007; Zhang et al., 2012). The increased capacity of female CD4^+^ T cells to produce IFNγ was associated with lower levels of PPARα and elevated levels of PPARγ compared to male CD4^+^ T cells. In males, endogenous androgens increased PPARα and reduced PPARγ levels in CD4^+^ T cells, suggesting that sex differences in CD4^+^ T cell functional responses are regulated by sex hormones *in vivo*.

Biallelic expression of X-linked genes that escape XCI also impacts T cell function. *Cxcr3* is located on the X chromosome, and during inflammation is upregulated on macrophages, dendritic cells, and CD8^+^ and CD4^+^ T cells, promoting their migration to sites of infection (Oghumu et al., 2019). The *Cxcr3* gene escapes XCI, and biallelic expression of *Cxcr3* enhances the Th1 anti-viral response and is associated with increased CD69 expression and production of IFNγ and IL-2 (Wang et al., 2016; Oghumu et al., 2019). *CD40LG* (encoding CD40L) expressed by T cells is another X-linked gene reported to escape XCI (Wang et al., 2016; Sarmiento et al., 2019). CD40L promotes T cell survival and regulates B cell activation and differentiation. Stimulated CD3^+^ T cells from (46, XX) women and (47, XXY) KS men showed significantly more CD40L protein density and mRNA as compared to cells from (46, XY) men and (45, XO) TS women (Sarmiento et al., 2019). Biallelic expression of CD40L enhances the adaptive immune response, however it has primarily been studied in the context of autoimmunity (Sarmiento et al., 2019; Syrett and Anguera, 2019).

During respiratory virus infection, B cell production of antibodies is an important mechanism of virus neutralization and clearance (Lam and Baumgarth, 2019). In addition, generation of memory B cells capable of producing high affinity antiviral antibodies upon infection or vaccination limits viral load and pathology upon subsequent infection with homo- or heterosubtypic viruses (Dhakal et al., 2021; Ursin et al., 2022). Sex differences in antibody production, particularly after vaccination, have been well studied in small mammal models and humans. Females produce a larger quantity and repertoire of antibodies specific for IAV and SARS-CoV-2 proteins (Lorenzo et al., 2011; Fink et al., 2018; Voigt et al., 2019; Dhakal et al., 2021; Ursin et al., 2022). An elegant study showed that enhanced seroconversion of HA-inhibiting antibodies was positively associated with the levels of estradiol in both adult and aged women, and that treatment of gonadectomized mice with either estradiol (females) or testosterone (males) led to an increase or decrease in antibody response, respectively (Potluri et al., 2019). These data confirm findings from an earlier study that showed levels of testosterone negatively correlate to antibody responses in males after vaccination against IAV (Furman et al., 2014). Due to their expression of both ERɑ and ERβ (Hill et al., 2011), B cells are likely to be directly regulated by estrogens during viral infection.

Much of our information about mechanisms by which estrogens and ERα regulate B cells function comes from studies of female sex-biased autoimmune diseases such as systemic lupus erythematosus, in which B cells produce high levels of autoantibodies (Dodd and Menon, 2022). ERα acts to modulate BCR signaling and promote the loss of B-cell tolerance, specifically in females, in murine lupus models (Grimaldi et al., 2001; Hill et al., 2011; Graham et al., 2020). Other work showed that ER activity enhances activated B cell expression of AID (encoded by *Aicda*), which is required for class switch recombination and somatic hypermutation (Muramatsu et al., 2000; Mai et al., 2010). *Aicda* expression is induced by the synergistic binding of HOXC4 and NF-κB to regulatory elements (Park et al., 2009), and ERɑ promotes this interaction by binding to the *HoxC4* promoter (Mai et al., 2010). In addition, AID expression can be silenced through multiple mechanisms including binding of miR-26a to the 3′UTR of the *Aicda* mRNA. Using both global (*Esr1* ^*−/−*^) or B cell specific *Esr1* deficient (*Aicda-cre-Esr1* ^*−/−*^) mice, researchers determined that ERɑ downregulated the expression of miR-26a through both direct and indirect binding to the genes responsible for miR-26a expression. The downregulation of miR-26a decreased AID silencing, thereby increasing somatic hypermutation (Casali et al., 2020). This mechanism may contribute to the enhanced antibody production seen in females in infection or vaccination.

B cells also display sex differences based upon their sex chromosome complement. Maintenance of *XIST* in naïve T and B cells is altered until they are activated, allowing for greater biallelic expression of some X-linked genes (Wang et al., 2016). One such gene is *CXORF21*, which encodes for the protein TASL (Heinz et al., 2020). TASL acts synergistically with TLR7, enhancing the downstream immune responses of TLR7 signaling and type 1 IFN production (Harris et al., 2019; Odhams et al., 2019). In B cells, TLR7 signaling promotes isotype switching, maturation, and antibody production (Jego et al., 2003). Biallelic expression of TLR7 in B cells is associated with greater antiviral antibody responses. In mice, vaccination with whole or subunit inactivated IAV was linked to greater expression of *Tlr7* in females as compared to males, leading to a higher quantity and quality of IAV-specific antibodies (Fink et al., 2018). Human studies also show that after vaccination with the trivalent inactivated influenza vaccine, females of reproductive age generate higher antibody titers than males (Potluri et al., 2019). Elevated expression of TLR7 is also linked to the production of autoantibodies in autoimmune diseases, such as systemic lupus erythematosus (Souyris et al., 2018).

## Lessons from regulatory and type 2 immunity

Often studied in the context of type 2 inflammation, such as asthma or helminth infections, regulatory T cells (Tregs) and innate type 2 lymphocytes (ILC2s) contribute to viral infections by promoting resolution and repair. In response to viral insult, infected epithelial cells release the alarmin IL-33 (Le Goffic et al., 2011), which in turn activates ILC2s and Tregs [reviewed in (Liew et al., 2016)]. Activated ILC2s secrete large amounts of IL-5, IL-4, and IL-13 (Kabata et al., 2018) that activate other immune cells, whereas Tregs most notably release high amounts of IL-10 that quell the inflammatory environment (Schmidt et al., 2012). Several reports in mouse models of asthma have indicated that both cell-intrinsic and extrinsic signaling through sex steroid receptors may regulate the function and/or development of both cell types.

ILC2s express high levels of *Ar* mRNA with little to no expression of *Esr1* or *Esr2* (Heng et al., 2008; Laffont et al., 2017), with the exception of those in the uterus (Bartemes et al., 2018). Androgens negatively regulate the transition of ILC precursors in the bone marrow to mature ILC2s in the lungs of male mice, which correlates to a two-fold increase in total numbers in the female lung compared to age matched males (Kadel et al., 2018). This difference in ILC2 numbers is mirrored in the blood of asthmatic patients (Cephus et al., 2017). Orchiectomy of male mice abolished the sex differences observed in asthma (Laffont et al., 2017), and androgen treatment of female mice after ovariectomy resulted in ILC2 numbers and functional responses typical of intact males (Blanquart et al., 2022). While these studies show that ILC2 development and number are influenced by sex steroids, mice bearing AR deficiency, specifically in ILC2s, will help delineate the cell intrinsic mechanisms that drive these phenotypes.

Tregs were reported to express quite low levels of *Ar, Esr1* and *Esr2* RNA (Heng et al., 2008), yet studies show evidence for cell-intrinsic ER or AR activity in Tregs. AR signaling reduces the number of pro-inflammatory Tregs in asthmatic male mice by decreasing their expression of IL-33R and activation by IL-33 (Gandhi et al., 2022). In contrast, physiological and pharmaceutical levels of E2 increase expansion of natural Tregs and induce the expression of FOXP3 in CD4^+^CD25^–^ T cells in an ERα-dependent manner (Polanczyk et al., 2004; Tai et al., 2008). E2 acting *via* ERβ augments Treg expression of FOXP3, CD25 and GATA3, and E2-exposed Tregs promote resolution of bacterial pneumonia (Xiong et al., 2021). As reduction of inflammation is essential for recovery from viral infections, sex steroid regulation of Treg numbers and function may contribute to the sex differences observed in immunopathology following viral infection.

Taken together, these data highlight a role for the sex steroid regulation of Treg and ILC2 numbers and function in the respiratory tract. Notably, both ILC2s and Tregs produce high levels of the growth factor amphiregulin (Arpaia et al., 2015; Kabata et al., 2018), and loss of this function exacerbates immunopathology and delays tissue repair in murine IAV infection (Monticelli et al., 2011; Arpaia et al., 2015). IAV infected males produce higher levels of amphiregulin, which correlates with improved pulmonary function compared to females in the recovery phase (Vermillion et al., 2018b). While treatment of gonadectomized males with testosterone improved IAV outcomes, direct regulation of amphiregulin by AR activity in ILC2s or Tregs was not reported. Androgens have been shown to increase the number of epithelial cells that release IL-33 in the visceral adipose tissue and increase the number of immunosuppressive Tregs in males (Vasanthakumar et al., 2020), consistent with the elevated numbers of stable Tregs observed in males with asthma (Gandhi et al., 2022). In contrast, lungs in naïve 8 week old females were shown to contain higher amounts of IL-33 compared to male lungs (Matha et al., 2019). These AR-mediated mechanisms may contribute to the protection observed in males after IAV infection (Vom Steeg et al., 2019; Vom Steeg et al., 2020). As Tregs are required for the development of CD8^+^ memory T cells (de Goer de Herve et al., 2012), Treg expansion promoted by estradiol may lead to the enhanced protection against secondary viral infections observed in females (Ursin and Klein, 2021).

Male sex and AR regulation of type 2 immunity has implications for viral infection and secondary links to asthma susceptibility that differs before and after puberty. In a murine model of neonatal RSV infection, female pups showed better virus control with higher levels of IFNβ not seen in male pups (Malinczak et al., 2019). This led to persistent immune cell skewing in male offspring, including elevated Th2 and Th17 cells, ILC2s, and type 2 cytokines such as IL-33 and TSLP that correlated with exacerbation of allergic responses, which are often preferentially observed in young boys. However, in murine models of asthma after sexual maturity, AR activity decreases effector Th2 and Th17 cells, which correlates with epidemiological data showing that asthma propensity is decreased in males after adolescence. RNA-sequencing analyses of AR-deficient T cells show an increase in the production of type 2 cytokines in males with little effect on females, further highlighting an anti-inflammatory role of androgens in males (Ejima et al., 2022). Mouse models of house dust mite-induced asthma show that cell intrinsic AR activity attenuated IL-17A^+^ Th17 cells, leading to a decrease in neutrophil infiltration, while endogenous androgens indirectly decreased the number of pro-inflammatory IL-13^+^ Th2 cells by suppressing IL-4 production (Fuseini et al., 2018). Future research will determine if common pathways are activated or attenuated by intrinsic AR activity in Th1 cells prominent in IFN-centered respiratory virus infections and/or in Th2 and Th17 cells important in asthma and extracellular bacterial infections.

## Conclusion

Sex steroids and X chromosome complement act in tandem or independently to mediate the sex differences seen in human disease and animal models (Figure 2). Herein, we have summarized evidence that sex steroids bound to their receptors and/or gene dosage on the X chromosome regulate immune responses in respiratory virus infection in humans and animal models. We have discussed how our understanding of immune mechanisms in infection may be informed by studies of sex differences in inflammatory pathways in autoimmune and other immune-mediated diseases. Taken together, these data reinforce the idea that multi-faceted immune responses to infection may be governed at various points by factors related to sex differences. For example, AR signaling may promote resolution of immune responses by attenuating T cell responses, while ER signaling may promote type I IFN production by innate cells. Secondly, a sex difference in response to or outcome of infection may be due to the presence or absence of sex-specific factors. For example, a particular pathway outcome in males may be due either to direct effects of AR signaling or the absence of robust ER signaling.

Ideally, an increased understanding of sex specific mechanisms underlying sex differences in pulmonary immunity would lead to improved treatment and precision medicine options for respiratory infection (Stachenfeld and Mazure, 2022). In the COVID-19 pandemic, observations of a male bias in mortality led to clinical trials involving estrogen or selective ER modulator administration or androgen blockade. To date, these clinical trials for COVID-19 have been inconclusive, and the field currently lacks consensus as to whether disparate sex steroid levels drive susceptibility or infection outcomes in the human population [reviewed in (Lott et al., 2022)]. Human studies that ascertain hormonal and chromosomal sex will benefit the field, as valuable data may be lost when sex steroid levels and sex chromosome complement are not considered and/or assumed to match overt gender identity. The challenge is to understand more fully how cell intrinsic sex steroid receptor expression and X chromosome complement regulate the epigenome, transcriptome, and proteome in immune cells, either prior to or during infection. This information will increase our understanding of the sex disparate pathways underlying immune function in antiviral responses.
